# Tedizolid Targets AQP9-JAK/STAT Axis to Suppress Metastatic Progression in Clear Cell Renal Cell Carcinoma: Mechanism and Therapeutic Implications

**DOI:** 10.3390/ijms27104234

**Published:** 2026-05-09

**Authors:** Kexin Qu, Tianya Zhang, Rui Wang, Yingwei Bi, Jiacheng Jin, Yuxin Liu, Bolin Yi, Liang Zhu, Jianbo Wang

**Affiliations:** 1 Department of Urology, The First Affiliated Hospital of Dalian Medical University, Dalian 116011, China; qkx3141@163.com (K.Q.); leynad1214@hotmail.com (R.W.); biyingwei_@outlook.com (Y.B.); jiachengjin.med@gmail.com (J.J.); 19845733936@163.com (Y.L.); yibl@dmu.edu.cn (B.Y.); 2 Department of Clinical Laboratory, The First Affiliated Hospital of Dalian Medical University, Dalian 116011, China; zhangty01@dmu.edu.cn; 3 Advanced Institute for Medical Sciences, Dalian Medical University, Dalian 116011, China

**Keywords:** Aquaporin-9, clear cell renal cell carcinoma, metastasis, JAK/STAT pathway, drug repurposing, tedizolid

## Abstract

Metastasis is a primary driver of poor outcomes in clear cell renal cell carcinoma (ccRCC), yet the role of Aquaporin-9 (AQP9) in this process remains unclear. This study aimed to investigate the function, clinical significance, and therapeutic potential of AQP9 in ccRCC. AQP9 expression was analyzed using TCGA data and validated in human tissues and cell lines via Western blot. Functional assays assessed malignant behaviors, while bioinformatics and rescue experiments explored the involvement of the JAK/STAT pathway and epithelial–mesenchymal transition (EMT). Virtual screening, molecular docking, and cellular thermal shift assays (CETSAs) were employed to identify Tedizolid as a potential AQP9 inhibitor, followed by functional validation in vitro and in a xenograft model. AQP9 was significantly upregulated in ccRCC and associated with poor prognosis. The knockdown of AQP9 suppressed proliferation, migration, invasion, and EMT, whereas its overexpression promoted these effects by activating the JAK/STAT pathway. Tedizolid bound directly to AQP9, inhibited cell viability, reversed AQP9-induced malignant phenotypes, and suppressed JAK/STAT signaling both in vitro and in vivo. In conclusion, AQP9 promotes ccRCC metastasis through the JAK/STAT-EMT axis and represents a potential prognostic biomarker and therapeutic target. Tedizolid, identified as a novel AQP9 inhibitor, offers a promising repurposed strategy for ccRCC treatment.

## 1. Introduction

Renal cell carcinoma (RCC), a diverse group of cancers derived from renal tubular epithelial cells, represents the third most prevalent malignancy of the urinary system, following prostate and bladder cancers [1], and its global incidence continues to rise [2]. Among all RCC’s pathological types, clear cell RCC (ccRCC) has the biggest proportion, approximately occupying up to 75% of RCCs [3]. Although early-stage ccRCC can generally achieve a favorable prognosis through surgical treatment, about 20–40% of patients with localized tumors eventually experience distant metastases post-surgery [4]. Furthermore, around 17% of ccRCC patients have already developed metastasis upon initial diagnosis; the five-year survival rate here is merely 12%, which shows that metastasis is one of the reasons why treatment fails and patients die [5]. Over recent years, immunotherapy and targeted therapy have been adopted as routine treatments; they have helped improve the outcome of patients with advanced/metastatic ccRCC. Nevertheless, due to cancer’s heterogeneity, drug resistance as well as treatment toxicity, we still face great difficulties now; hence new kinds of drugs need to be urgently discovered.

Tumor metastasis is a complex biological process involving multiple factors, and epithelial–mesenchymal transition (EMT) serves as a critical step. During EMT, epithelial cells lose typical epithelial characteristics such as polarity and cell–cell adhesion, and acquire mesenchymal properties such as enhanced invasion, migration, and resistance to apoptosis [6]. In ccRCC cases, the occurrence of EMT can predict worse prognosis since it is always found along with higher grade tumors and a more advanced disease stage [7,8]. It has already been proved before that some signal pathways like the Wnt/β-catenin pathway, TGF-β pathway and PI3K/Akt pathway can regulate EMT [9,10,11]. In recent years, people have discovered more evidence showing us that the JAK/STAT pathway (especially constant activation of STAT3) may also play an indispensable role in controlling the EMT of various cancers like lung adenocarcinoma and gastric cancer [12,13,14]. As a transcription factor, activated STAT3 translocates to the nucleus and directly binds to the promoter regions of EMT-related transcription factors such as SNAIL and SLUG, promoting their expression. STAT3 also upregulates matrix metalloproteinases including MMP1, MMP2, MMP9, and MMP10, which degrade the extracellular matrix (ECM), thereby promoting tumor cell migration and invasion [15]. Although abnormal activation of the JAK/STAT pathway in ccRCC has been reported [16,17], the full spectrum of upstream signals initiating this pathway has not yet been fully elucidated. Therefore, identifying the specific upstream regulators of the JAK/STAT pathway in ccRCC is important for understanding its metastasis mechanism.

Aquaporin is a class of transmembrane protein that helps water and certain small solutes go across cell membranes [18]. In addition to keeping cell size constant and internal milieu stable, new research suggests that AQPs also participate in some kind of cellular activity, like cell migration, proliferation, and energy metabolism. They may also contribute to cancer development and progression [19]. AQP9 is one member of the aquaporin family and can transport glycerol, urea, and other small uncharged molecules [20]. Increasing evidence indicates that AQP9 contributes to tumor formation. In prostate cancer, AQP9 promotes proliferation, metastasis, and resistance to apoptosis in hormone-independent cells via the ERK pathway [21]. Meanwhile in breast cancer, neutrophils elevate the expression of AQP9. Then, activated STAT3 is stimulated by AQP9, which subsequently drives cancer cell growth [22]. Interestingly, however, AQP9 seems able to inhibit tumor progression when dealing with liver cancer and laryngeal cancer [23,24]. Concerning clear cell renal cell carcinoma, bioinformatics analysis and clinical evidence have implied that AQP9 is likely to be a predictive indicator for patients’ prognosis [25,26]. One study also showed that miR-532 may regulate the tumor-promoting effect of AQP9 in ccRCC [27]. But, up until now, we still do not fully understand what exactly makes AQP9 act upon ccRCC. Therefore, more investigations need to be carried out on this topic.

The treatment of advanced-stage ccRCCs has now entered the phase of combining the targeted approach and immunotherapy. However, tumor heterogeneity, drug resistance, as well as toxicity still pose big challenges. Therefore, new anti-ccRCC drugs need to be developed. Nevertheless, starting from scratch will take quite some time and requires huge financial cost, while drug repurposing can provide us with a relatively fast and cheap way out. In addition, the known safety profile of these drugs could make them more acceptable for drug development against cancer [28].

In this study, firstly, we proved that AQP9 is potentially one of the promising therapeutic targets in ccRCC. AQP9 can cause EMT via the JAK/STAT pathway in ccRCC cell lines, which contributes to increased capability of cell migration and invasion. Then, we found out that Tedizolid (which has been approved for use in skin infections as an antibiotic [29]) could block the expression of AQP9 and affect its function. Cell experiments and animal experiments indicate that Tedizolid can inhibit tumor growth and dissemination because of the inhibition of AQP9. Therefore, we consider that Tedizolid might be a new potential medicine to treat ccRCC.

## 2. Results

### 2.1. AQP9 Was Upregulated in ccRCC and Associated with Poor Prognosis

**Figure 1:**
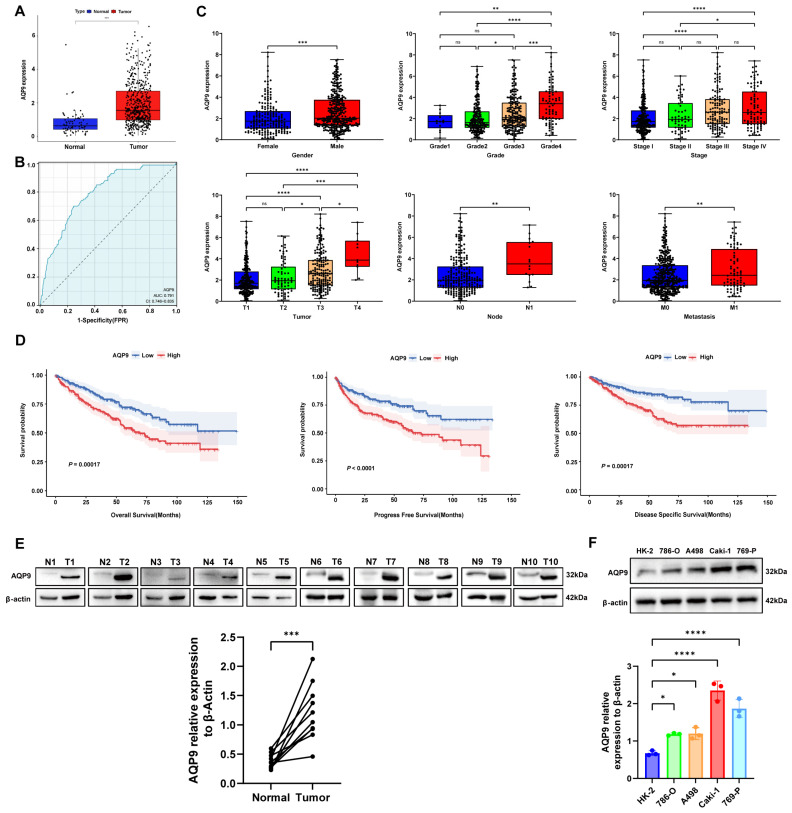
The high expression of AQP9 in ccRCC is associated with poor prognosis. (**A**) Expression levels of AQP9 in ccRCC and normal kidney tissues from the TCGA database. (**B**) ROC curve analysis shows the ability of AQP9 to distinguish patients with ccRCC from non-cancerous individuals. The area under the curve (AUC) is 0.791 (95% CI: 0.746–0.835). The diagonal dashed line indicates random prediction. (**C**) The correlation between AQP9 expression and various clinical characteristics of ccRCC patients in the TCGA database. (**D**) Kaplan–Meier survival analysis showing the impact of AQP9 expression on the overall survival (OS), progression-free survival (PFS), and disease-specific survival (DSS) of ccRCC patients from the TCGA database. (**E**) Western blot analysis of AQP9 protein expression in 10 paired ccRCC tumors (T) and adjacent non-tumor tissues (N). The AQP9 band migrated at approximately 32 kDa in T tissues and approximately 35 kDa in N tissues. Each pair of T and N from the same patient is connected by a line. β-actin served as a loading control. (**F**) Western blot analysis of AQP9 protein expression in the normal renal tubular epithelial cell line HK2 and four ccRCC cell lines (786-O, A498, Caki-1, and 769-P). β-actin served as a loading control. The data are presented as mean ± SD. * *p* < 0.05, ** *p* < 0.01, *** *p* < 0.001, **** *p* < 0.0001.

We first identified that AQP9 expression was significantly increased in ccRCC tissues relative to adjacent normal tissues through the TCGA database (Figure 1A). Subsequently, we evaluated the diagnostic potential of AQP9 for ccRCC using receiver operating characteristic (ROC) curves, which revealed an AUC value of 0.791 (95% CI: 0.746–0.835), suggesting that AQP9 could serve as a potential target and biomarker for ccRCC (Figure 1B). We then analyzed the relationship between AQP9 expression and various clinical features of ccRCC patients, such as sex, clinical stage, and pathological grade, and plotted box plots (Figure 1C). Our analysis showed that AQP9 expression increased with higher clinical stage and grade of ccRCC, suggesting a significant link between AQP9 and ccRCC malignancy. Subsequently, survival analyses comparing high and low AQP9 expression groups were performed, with survival curves illustrating that elevated AQP9 levels were associated with significantly reduced overall survival (OS), progression-free survival (PFS), and disease-specific survival (DSS) (Figure 1D), indicating that high AQP9 expression might predict poor prognosis. Multivariate Cox regression analyses for OS, PFS, and DSS conducted on ccRCC cases from the TCGA database (Table 1, Table 2 and Table 3) identified AQP9 as an independent factor of prognosis across all three survival models. A Western blot analysis conducted on 10 paired ccRCC and adjacent normal tissue samples demonstrated a significant upregulation of AQP9 protein in the tumor tissues (Figure 1E). Of note, the AQP9 band migrated at approximately 32 kDa in the tumor tissues and at approximately 35 kDa in the adjacent normal tissues. This reproducible migration difference likely reflects the differential N-glycosylation of AQP9 between normal and malignant renal tissues, as AQP9 is known to undergo N-linked glycosylation and aberrant glycosylation is a recognized feature of ccRCC [30,31,32]. Moreover, a Western blot analysis of AQP9 expression in the normal human renal tubular epithelial cell line HK2 and four ccRCC cell lines (786-O, A498, Caki-1, and 769-P) showed markedly higher AQP9 levels in all the ccRCC cell lines compared to the HK2 cells (Figure 1F). Collectively, these results indicated that AQP9 was critically involved in ccRCC development and progression and represented a key prognostic biomarker for this malignancy.

### 2.2. AQP9 Promoted the Proliferation of ccRCC Cells

**Figure 2:**
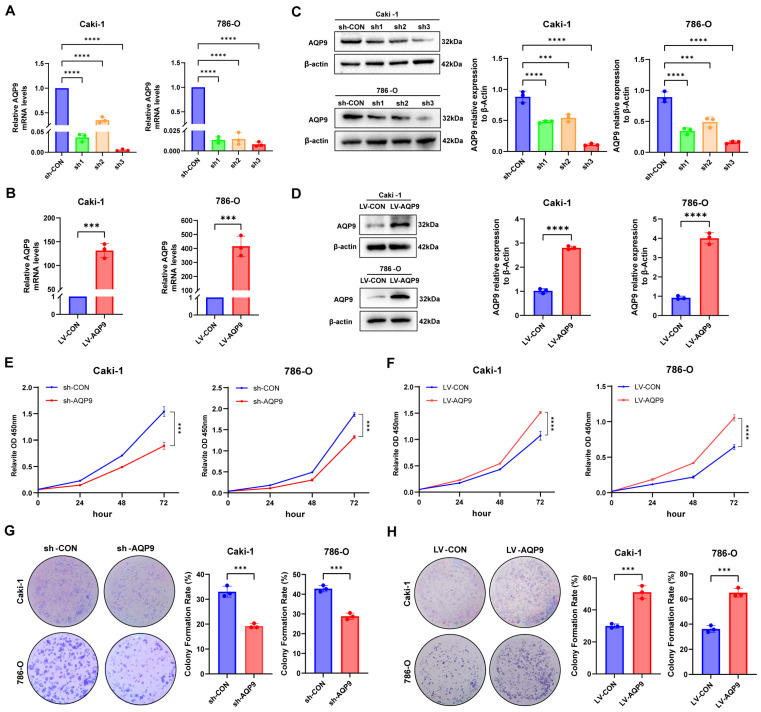
AQP9 promotes the proliferation and colony formation of ccRCC cells. (**A**,**B**) qRT-PCR analysis of AQP9 mRNA levels in Caki-1 and 786-O cells transfected with sh-CON, sh-AQP9, LV-CON, or LV-AQP9. β-actin was used for normalization. (**C**,**D**) A Western blot analysis of AQP9 protein expression in Caki-1 and 786-O cells transfected with sh-CON, sh-AQP9, LV-CON, or LV-AQP9. β-actin served as a loading control. (**E**,**F**) A CCK-8 assay assessing the viability of Caki-1 and 786-O cells following AQP9 knockdown or overexpression. (**G**,**H**) A colony formation assay evaluating the clonogenic capacity of Caki-1 and 786-O cells following AQP9 knockdown or overexpression. The data are presented as mean ± SD. *** *p* < 0.001, **** *p* < 0.0001.

To investigate the specific role of AQP9 in the progression of ccRCC, we constructed AQP9-knockdown and AQP9-overexpression stable cell lines of 786-O and Caki-1, and confirmed transfection efficiency by qRT-PCR and Western blot. The results showed that AQP9 was effectively regulated at both the mRNA (Figure 2A,B) and protein (Figure 2C,D) levels in both cell lines. Furthermore, in both cell lines, sh-AQP9 3 exhibited the most significant knockdown efficiency of AQP9; thus, sh-AQP9 3 was used for subsequent functional studies. At the cellular functional level, our findings demonstrated that the knockdown of AQP9 significantly suppressed the proliferation and clonogenic ability of the ccRCC cells (Figure 2E,G), whereas its overexpression produced the opposite effect (Figure 2F,H). These results indicated that AQP9 facilitated the proliferation and clonogenic ability of the ccRCC cells, thereby exerting a pro-cancer effect in ccRCC.

### 2.3. AQP9 Promoted the Migration, Invasion, and Epithelial–Mesenchymal Transition (EMT) Process in ccRCC Cells

**Figure 3:**
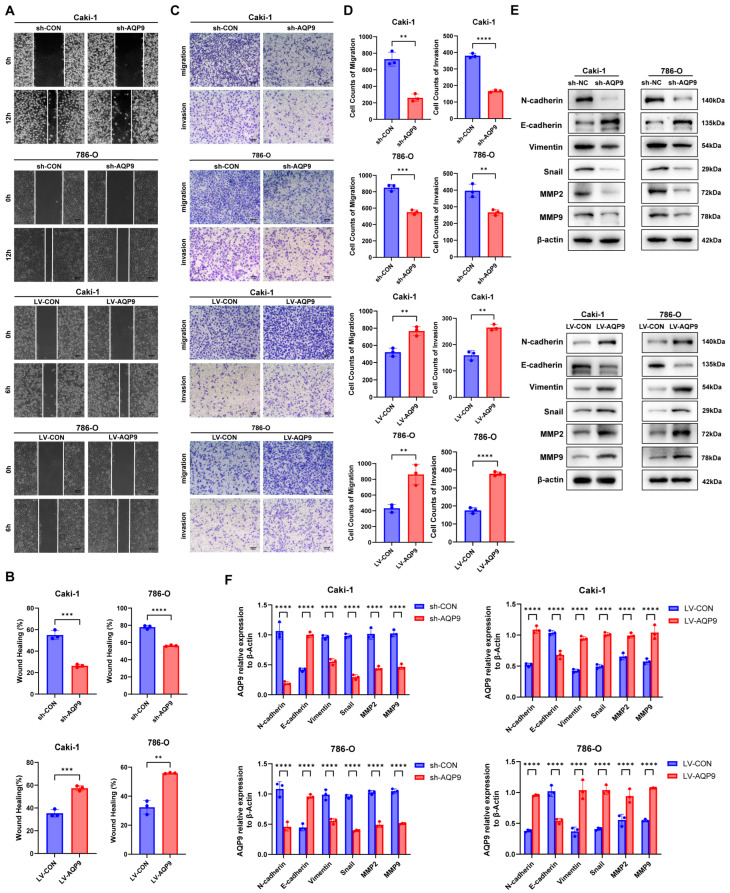
AQP9 promotes migration, invasion, and EMT in ccRCC cells. (**A**,**B**) A wound healing assay showing the migratory ability of Caki-1 and 786-O cells after AQP9 knockdown or overexpression. (**C**,**D**) Transwell migration and invasion assays of Caki-1 and 786-O cells after AQP9 knockdown or overexpression. (**E**,**F**) A Western blot analysis of EMT-related markers (N-cadherin, E-cadherin, Vimentin, Snail, MMP2, and MMP9) in Caki-1 and 786-O cells after AQP9 knockdown or overexpression. β-actin served as a loading control. The data are presented as mean ± SD. ** *p* < 0.01, *** *p* < 0.001, **** *p* < 0.0001.

To determine the role of AQP9 in ccRCC metastasis, we performed a wound healing assay and Transwell migration and invasion assays to analyze whether AQP9 affected cell migratory and invasive capabilities. Silencing AQP9 inhibited cell migration and wound healing abilities, whereas AQP9 overexpression accelerated this process (Figure 3A,B). Consistently, the Transwell assays showed that AQP9 knockdown markedly reduced ccRCC cell migration and invasion, while its overexpression promoted these capabilities (Figure 3C,D). Furthermore, a Western blot analysis of key EMT-related proteins demonstrated that AQP9 knockdown led to the upregulation of the epithelial marker E-cadherin and the downregulation of mesenchymal markers such as N-cadherin, Vimentin, the EMT transcription factor Snail, and matrix metalloproteinases MMP2 and MMP9. Conversely, AQP9 overexpression produced opposite effects (Figure 3E,F). In conclusion, AQP9 promoted migration and invasion by inducing EMT in ccRCC cells.

### 2.4. AQP9 Promoted the Activation of the JAK/STAT Pathway in ccRCC Cells

**Figure 4:**
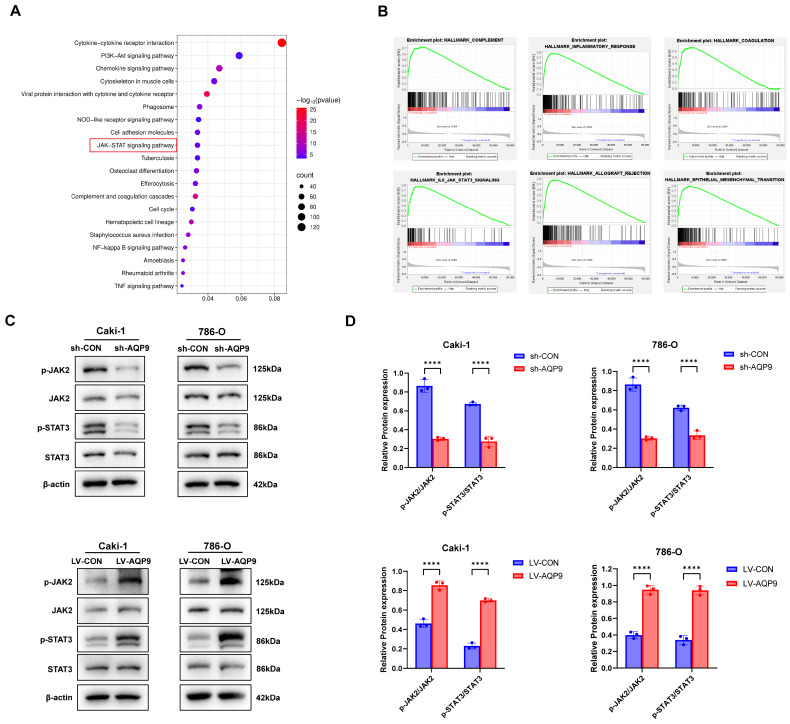
AQP9 activates the JAK/STAT signaling pathway in ccRCC cells. (**A**) A KEGG pathway enrichment analysis of genes co-expressed with AQP9. (**B**) A GSEA plot showing significant enrichment of the JAK/STAT signaling pathway gene set in tumors with high AQP9 expression. (**C**,**D**) A Western blot analysis of key JAK/STAT pathway proteins (JAK2, STAT3, and their phosphorylated forms p-JAK2 and p-STAT3) in Caki-1 and 786-O cells after AQP9 knockdown or overexpression. β-actin served as a loading control. The data are presented as mean ± SD. **** *p* < 0.0001.

In order to investigate what particular kind of molecular mechanism AQP9 acts through to promote the malignant phenotype of ccRCC, we performed a KEGG and GSEA pathway enrichment analysis. We discovered that AQP9 has very strong relevance with a certain pathway—the JAK/STAT pathway (Figure 4A,B). Therefore, we tested the expression level of several important factors in this JAK/STAT pathway (JAK2, STAT3, p-JAK2, p-STAT3) through Western blot. And the findings were: Compared with the controls, when the AQP9 gene is silenced, it will suppress the activation status of the JAK/STAT pathway. However, when AQP9 is overexpressed, the activation status of this pathway is significantly increased (Figure 4C,D). These results suggest that the JAK/STAT pathway may mediate the oncogenic effects of AQP9 in ccRCC.

### 2.5. AQP9 Regulated ccRCC EMT Through the JAK/STAT Pathway

**Figure 5:**
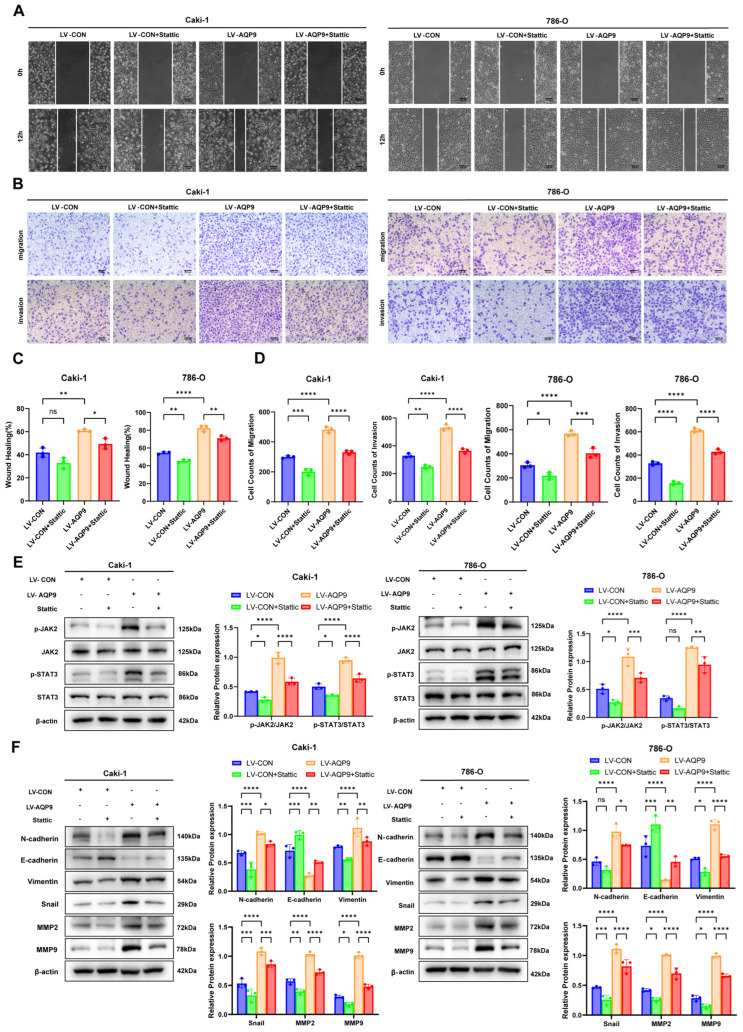
AQP9 regulates EMT in ccRCC via the JAK/STAT pathway. (**A**,**C**) A wound healing assay showing that the STAT3 inhibitor Stattic attenuates the enhanced migration of ccRCC cells induced by AQP9 overexpression. (**B**,**D**) Transwell migration and invasion assays showing that Stattic attenuates the enhanced migration and invasion of ccRCC cells induced by AQP9 overexpression. (**E**) A Western blot analysis showing that Stattic reverses the activation of the JAK2/STAT3 pathway induced by AQP9 overexpression. (**F**) A Western blot analysis showing that Stattic reverses the promotion of EMT markers induced by AQP9 overexpression. The data are presented as mean ± SD. * *p* < 0.05, ** *p* < 0.01, *** *p* < 0.001, **** *p* < 0.0001.

Based on previous research, we noted that the JAK/STAT pathway, particularly the sustained activation of STAT3, plays a crucial role in the EMT process of various malignant tumors [12,13,14]. Therefore, we hypothesized that the JAK/STAT pathway might be a key mechanism through which AQP9 affects the metastatic ability of ccRCC. To verify this hypothesis, we introduced the JAK/STAT pathway inhibitor Stattic (10 μM) into our experiments. Through scratch assays and Transwell migration and invasion assays, we found that in two ccRCC cell lines, Stattic partially inhibited the enhanced migration and invasion induced by AQP9 overexpression, and also exerted a certain inhibitory effect on migration and invasion in the control group (Figure 5A–D). We then confirmed the inhibitory influence of Stattic on the JAK/STAT pathway using Western blot and found that it significantly suppressed the JAK/STAT pathway activation triggered by AQP9 overexpression (Figure 5E). Furthermore, we examined the changes in EMT markers and found that Stattic partially reversed the changes in EMT markers caused by AQP9 overexpression (Figure 5F). These results suggest that AQP9 regulates the EMT process through the JAK/STAT pathway, thereby promoting ccRCC migration and invasion.

### 2.6. Identification and Validation of Tedizolid as a Potential AQP9 Inhibitor

**Figure 6:**
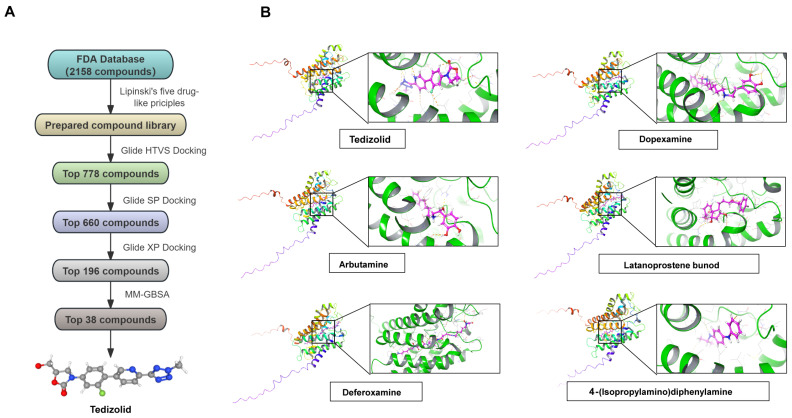
The identification of Tedizolid as a potential AQP9-targeting compound via virtual screening. (**A**) The workflow of the virtual screening process for identifying AQP9 binders from an FDA-approved drug library. (**B**) The predicted binding poses and calculated binding free energies (MM/GBSA, kcal/mol) of the top six candidate compounds docked into the AQP9 structure.

**Figure 7:**
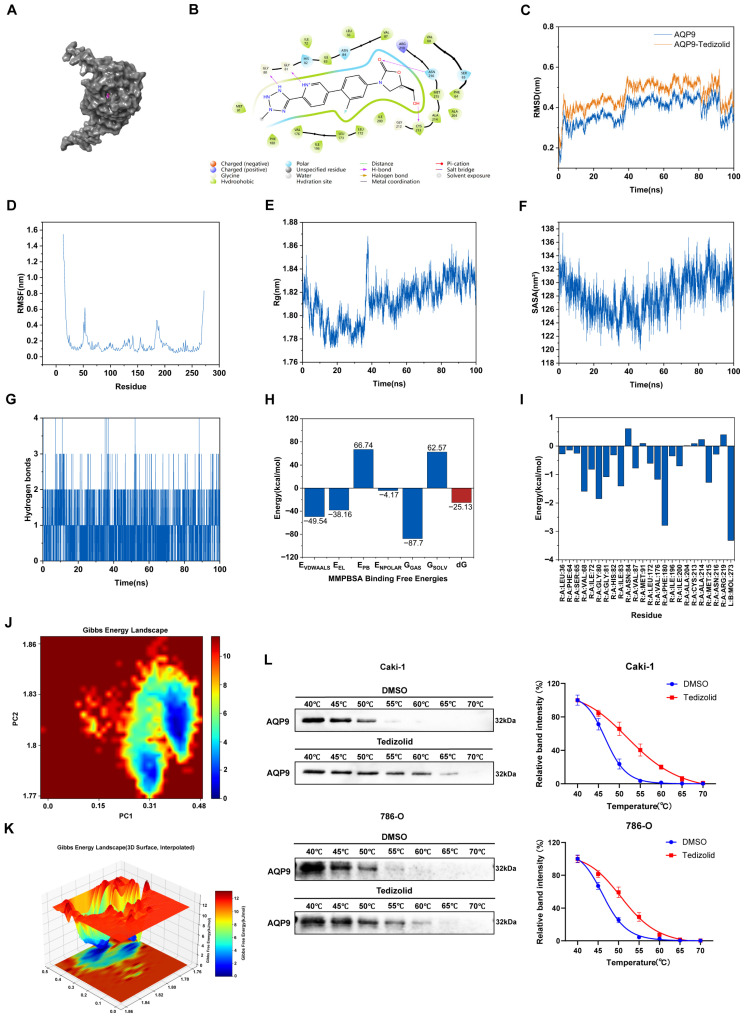
Molecular dynamics simulations validate the stable binding of Tedizolid to AQP9. (**A**) The overall structure of AQP9 with Tedizolid (green) bound in the central pore. (**B**) A two-dimensional ligand interaction diagram of Tedizolid with key AQP9 residues. (**C**) The RMSD of the AQP9–Tedizolid complex during the 100 ns simulation. (**D**) The RMSF per residue of AQP9 during the simulation. (**E**) The Rg of the AQP9 protein throughout the simulation. (**F**) The SASA of the AQP9 protein throughout the simulation. (**G**) The number of hydrogen bonds between Tedizolid and AQP9 during the simulation. (**H**) The calculated MM/PBSA binding free energy for the AQP9–Tedizolid complex. (**I**) A per-residue free energy decomposition plot for the AQP9–Tedizolid interaction. (**J**,**K**) Two- and three-dimensional free energy landscapes projected onto the first two principal components (PC1 and PC2). (**L**) A CETSA showing the thermal stabilization of AQP9 by Tedizolid.

Given the critical role of AQP9 in ccRCC progression, we aimed to evaluate its potential as a drug target. We screened 2158 FDA-approved compounds from DrugBank using structure-based virtual screening (Figure 6A). Based on MMGBSA binding free energies, we found six candidate compounds with good binding to AQP9 (Figure 6B). Tedizolid exhibited the most favorable binding free energy (−78.37 kcal/mol) and was chosen as the best candidate. Tedizolid fit tightly in the hydrophobic pocket of AQP9, forming four hydrogen bonds and 17 hydrophobic contacts (Figure 7A,B). Dopexamine ranked second with a binding free energy of −76.43 kcal/mol, interacting with AQP9 through four hydrogen bonds and 19 hydrophobic interactions. The other four compounds exhibited binding free energies between −66.82 and −73.65 kcal/mol. Given its superior binding performance, Tedizolid was selected for further dynamics simulations and functional experiments.

In order to evaluate the binding stability of the AQP9–Tedizolid complex, we performed a 100 ns MD simulation; then RMSD was analyzed, which suggested that the protein’s main-chain had been stabilized after about 40 ns (Figure 7C). Then we did an RMSF analysis, and found out that most residues showed low RMSF. There were only high RMSF values at the N-terminal part, indicating that this is a flexible region (Figure 7D). Rg was also stable after 40 ns. This means there is no big structural unfolding during the simulation (Figure 7E). In addition, SASA did not show any big fluctuations, therefore its confirmatory conformational stability can be proved in a solution environment (Figure 7F). And the hydrogen bonding analysis also showed stable interactions within the Tedizolid/AQP9 complex throughout the simulation (Figure 7G). In addition, MMPBSA calculation yielded a total binding free energy of −25.13 kcal/mol, indicating stable binding thermodynamics (Figure 7H). As for how much contribution each residue makes to the binding free energy, we can refer to Figure 7I. Residue energy decomposition identified PHE-180, GLY-80, and VAL-68 as major contributors to binding free energy. Conformational stabilities were evaluated by doing principal component analysis (PCA), and Gibbs free energy calculation. As shown in Figure 7J, there is a single low-energy valley (blue area), which indicates that it has very little conformational change within its own energy state and there is no obvious high-energy barrier dividing valleys. Then we constructed a three-dimensional free energy diagram that also proves that such conformations have good stability, which can correspond well to our docking result (Figure 7K). All the above results indicate that the complex of AQP9 and Tedizolid stays in a comparatively stable low-energy state, which provides us theoretical evidence for future function study.

In order to verify whether Tedizolid could bind to AQP9 in vivo, a CETSA was performed in Caki-1 and 786-O cells with the temperature ranging from 40 to 70 °C. Compared with DMSO controls, Tedizolid clearly increased soluble AQP9, as shown by a rightward shift in the melting curve (Figure 7L). Meanwhile, ΔTm of Tedizolid could increase by 6.27 °C (3.69–8.59) in Caki-1 cells and 4.81 °C (3.27–6.49) in 786-O cells, which meant that Tedizolid bound directly with AQP9 and enhanced the heat stability of this protein.

### 2.7. Tedizolid Inhibited the Progression of ccRCC by Inhibiting the AQP9-JAK/STAT Axis

**Figure 8:**
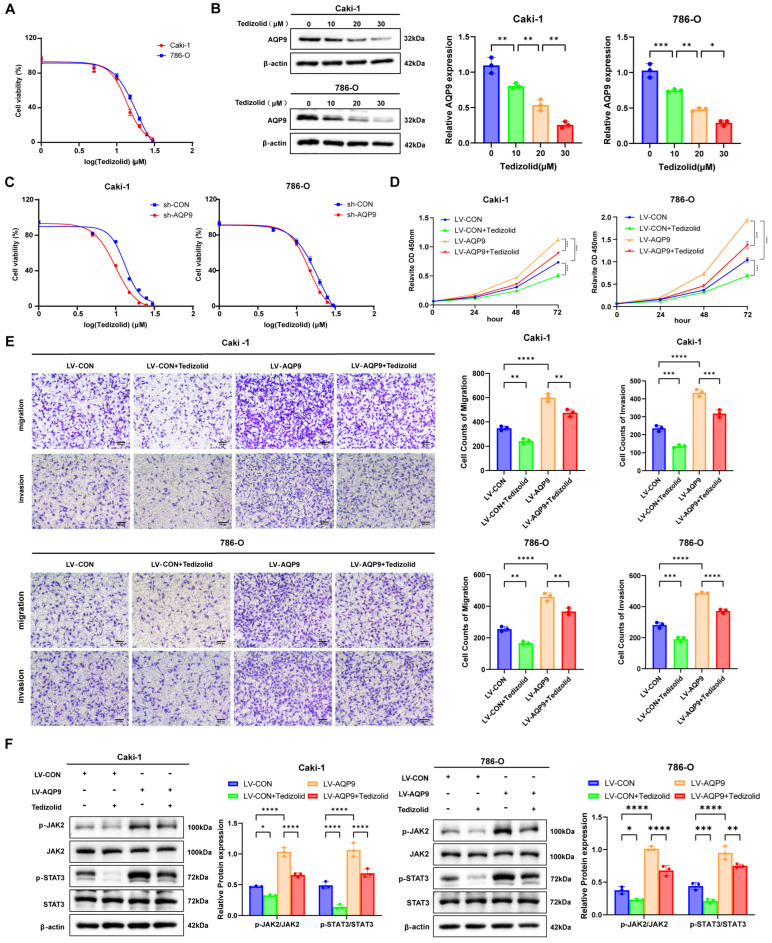
Tedizolid inhibits ccRCC progression by targeting AQP9. (**A**) A CCK-8 assay showing the dose-dependent inhibition of ccRCC cell viability by Tedizolid. The IC50 values are indicated. (**B**) A Western blot analysis showing the dose-dependent downregulation of the AQP9 protein by Tedizolid in the ccRCC cells. (**C**) A CCK-8 assay showing the increased sensitivity to Tedizolid upon AQP9 knockdown in the ccRCC cells. The IC50 values are indicated. (**D**) A CCK-8 assay showing that Tedizolid treatment reverses the enhanced proliferation induced by AQP9 overexpression. (**E**) Transwell assays showing that Tedizolid treatment reverses the enhanced migration and invasion induced by AQP9 overexpression. (**F**) A Western blot analysis showing that Tedizolid treatment reverses the activation of the JAK/STAT pathway induced by AQP9 overexpression. The data are presented as mean ± SD. * *p* < 0.05, ** *p* < 0.01, *** *p* < 0.001, **** *p* < 0.0001.

We used a CCK-8 assay to test how Tedizolid affects ccRCC cell survival in 786-O and Caki-1 cells. As illustrated in Figure 8A, Tedizolid decreased cell survival in a dose-dependent way in both cell lines. The IC50 was 13.48 (12.63–14.63) μM for the Caki-1 cells and 18.15 (16.37–22.53) μM for the 786-O cells. We then checked how Tedizolid affects AQP9 expression after 48 h at different doses (0, 10, 20, and 30 μM) by Western blot. Tedizolid reduced AQP9 protein in a dose-dependent manner in both cell lines (Figure 8B). The CCK-8 assay showed that AQP9 silencing lowered the IC50 from 13.17 (12.33–14.27) μM to 9.88 (9.49–10.28) μM in the Caki-1 cells, and from 19.04 (17.02–23.6) μM to 14.42 (13.85–15.1) μM in the 786-O cells. These results indicate that AQP9 knockdown sensitizes ccRCC cells to Tedizolid (Figure 8C). The CCK-8 assays (Figure 8D) and Transwell assays (Figure 8E) also showed that Tedizolid partly reversed the increased proliferation, migration, and invasion caused by AQP9 overexpression. Western blot showed that Tedizolid clearly blocked the JAK/STAT pathway activation caused by AQP9 overexpression (Figure 8F). These cell data show that Tedizolid suppresses the malignant features of ccRCC cells by blocking the AQP9-JAK/STAT axis.

### 2.8. In Vivo Experiments Confirmed That Tedizolid Inhibits ccRCC Progression by Targeting the AQP9-JAK/STAT Axis

**Figure 9:**
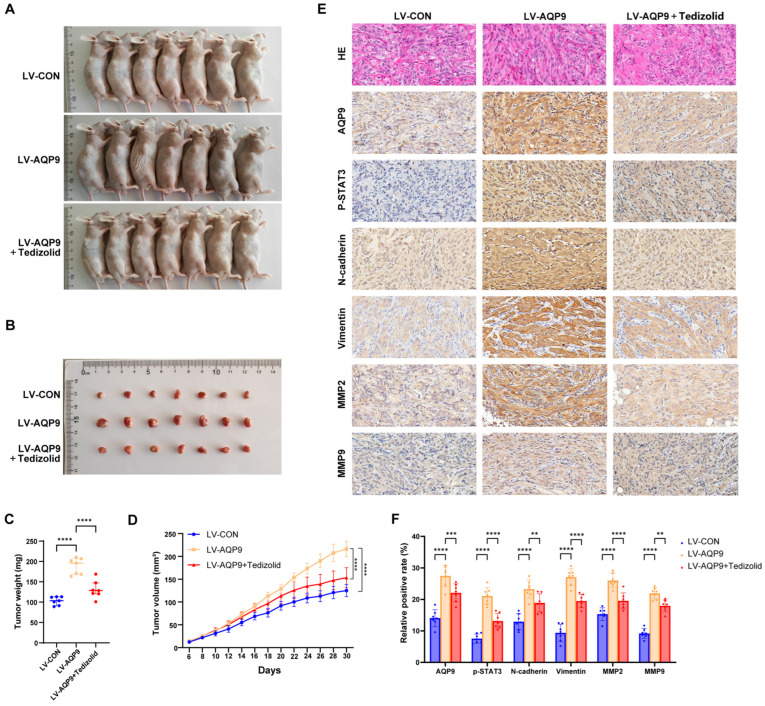
In vivo validation of Tedizolid inhibiting ccRCC progressions via the AQP9-JAK/STAT axis. (**A**,**B**) An overview of tumor formation in nude mice after the subcutaneous implantation of tumor cells (4 × 10^6^ cells per mouse) and the oral administration of Tedizolid (30 mg/kg/48 h, for 24 days). The images show representative tumors harvested at the experimental endpoint (Day 30). (**C**,**D**) The tumor weight and growth curves of the subcutaneous xenografts from the three groups. Compared to the LV-CON group, the LV-AQP9 group exhibited a significant increase in both tumor weight and volume. The administration of Tedizolid partially reversed this trend. The growth curves demonstrate that oral Tedizolid treatment (30 mg/kg/48 h) inhibited the increase in tumor volume starting from Day 12. (**E**,**F**) Representative H&E and IHC staining images showing the expression levels of AQP9, p-STAT3, N-cadherin, E-cadherin, Vimentin, MMP2, and MMP9 in the subcutaneous tumors from the three groups. The data are presented as mean ± SD. ** *p* < 0.01, *** *p* < 0.001, **** *p* < 0.0001.

To confirm the impact of AQP9 and Tedizolid on ccRCC growth in vivo, we used BALB/c nude mice. The mice were assigned into three groups (*n* = 7): (1) 786-O LV-CON, (2) 786-O LV-AQP9, and (3) 786-O LV-AQP9 + Tedizolid. We injected equal numbers of 786-O cells (with LV-CON or LV-AQP9) under the skin of each mouse. After tumors formed, the mice received treatment by oral gavage. At the termination of the study, all the mice were euthanized, and the subcutaneous tumors were resected for further analysis. The results indicated that AQP9 overexpression substantially increased tumor volume and weight, whereas treatment with Tedizolid partially reversed this trend (Figure 9A–D). Further H&E staining and immunohistochemical analysis (Figure 9E,F) revealed that AQP9 overexpression markedly upregulated the expression of AQP9, p-STAT3, N-cadherin, Vimentin, MMP2, and MMP9 in the ccRCC tissues. Treatment with Tedizolid partially reversed these protein expressions (Figure 9E,F). These in vivo findings robustly validate the conclusions drawn from our prior in vitro studies.

## 3. Discussion

In recent years, although advances in surgical techniques and the widespread use of combined targeted and immunotherapy have resulted in some improvement in the prognosis of patients with ccRCC, tumor metastasis and therapeutic resistance remain major challenges for patients with advanced ccRCC. Therefore, the identification of accurate and reliable biomarkers has become a major focus of ccRCC-related research. Previous studies have repeatedly demonstrated that AQP family members serve important functions in the initiation and progression of various malignancies [33,34,35]. AQP9, a significant member of the aquaporin family, facilitates the transport of water, glycerol, urea, and various other small molecules [20]. An earlier study showed that AQP9 is regulated by miR-532 and exerts a pro-tumorigenic effect in clear cell renal carcinoma [27]; however, the exact molecular mechanisms underlying AQP9’s role in ccRCC have yet to be completely clarified.

In this study, we systematically delineated, for the first time, the mechanism by which AQP9 exerts an oncogenic role in ccRCC, namely by activating the JAK/STAT pathway to promote EMT in ccRCC cells, thereby enhancing their malignant phenotype. Of greater translational relevance, by using hierarchical virtual screening and conducting in vitro and in vivo validation, we identified the FDA-approved antibiotic Tedizolid as an effective AQP9 inhibitor, thus providing a novel targeted agent for the treatment of ccRCC.

In this study, we firstly carried out our bioinformatics analysis and discovered that high AQP9 expression was independently correlated with a more advanced clinical stage, higher pathological grade, as well as shorter OS, PFS, and DSS. Therefore, we suggested that upregulated AQP9 expression might act as a risk factor for worse prognosis in ccRCC patients. Our findings are consistent with those reported by Wuqin Xu et al. in breast cancer, which indicated that high AQP9 expression was associated with poorer outcomes [22] and could promote cancer cell proliferation. In addition, several similar results have been described in other types of cancers, like prostate cancer [21], astrocytoma [36], and colorectal cancer [37] respectively. Notably, in hepatocellular carcinoma, AQP9 has repeatedly been consistently reported to exert a tumor-suppressive role [38,39,40,41], suggesting that the function of AQP9 may be highly tumor-type specific. Then, multivariate Cox regression analysis verified again that AQP9 was proved as an independent predictor of prognosis. And this result was the same as what Wen Hao Xu et al. drew through studying single-center clinical datasets [25]. Finally, we demonstrated high-level AQP9 expression both in the tissues and cells of ccRCC via Western blot, which provides compelling proof at the clinical level that AQP9 might be regarded as a potential biomarker of ccRCC. Notably, the AQP9 band consistently migrated at approximately 32 kDa in ccRCC tissues and cell lines, compared with approximately 35 kDa in adjacent normal tissues. This reproducible migration difference likely reflects the differential N-glycosylation of AQP9 between normal and malignant renal tissues. Gena et al. previously characterized AQP9 glycosylation in detail, identifying a 32 kDa unglycosylated core form (coreAQP9) and a 37–43 kDa N-glycosylated form (glycoAQP9), and demonstrated that the glycosylated form predominates at the plasma membrane in normal tissues [30]. In our data, the tumor band at 32 kDa corresponds to the coreAQP9 form, while the normal tissue band at 35 kDa falls between the core and fully glycosylated forms, suggesting partial basal glycosylation in normal kidney epithelium. The ~3 kDa shift is consistent with the mass of a single N-glycan moiety. The near-complete absence of glycosylated AQP9 in ccRCC is consistent with the immature N-glycan phenotype recently reported in ccRCC by glycomic profiling studies [31]. The functional significance of AQP9-specific glycosylation patterns in renal malignancy warrants dedicated investigation in future studies.

At the functional level, our in vitro experiments showed that silencing AQP9 reduced the proliferation rate of ccRCC cells; their ability to form colonies as well as their ability to migrate and invade were all reduced markedly. Meanwhile, the overexpression of AQP9 resulted in reverse effects. Those findings suggest AQP9 can play the role of oncogene in ccRCC. Then we examined several markers related to EMT and discovered the overexpression of AQP9 can activate EMT; in contrast, silencing AQP9 will produce opposite changes. Therefore, we concluded AQP9 may facilitate ccRCC migration and invasion by stimulating EMT. EMT is one of the main ways tumors obtain capability of migrating and invading [6]. Many studies have proved the AQP family’s regulatory impact on EMT: Wu S. et al. [42] reported that AQP1 could promote EMT in advanced breast cancer cells via the Wnt/β-catenin pathway; Jia Chen. et al. [43] found out that AQP3 influences gastric cancer metastasis through activating EMT; while He Z. et al. [44] found that AQP5 regulated EMT via the NF-κB pathway, thus promoting hepatic cell carcinoma metastasis. Thus, despite differences among AQP family members, the modulation of EMT to influence tumor metastasis may represent a common mechanism.

To explore the downstream mechanism of AQPs, we carried out a GSEA and KEGG pathway enrichment analysis which indicated that there was certain correlation between AQP9 and the JAK/STAT pathway. The JAK/STAT pathway has been proved to be associated with many kinds of cancers [45]; in particular, the continuous activation of STAT3 can drive tumor proliferation, EMT and immune evasion. Meanwhile it also plays a major role in inflammatory response [46]; our results showed that silencing AQP9 suppressed the phosphorylation of JAK2 and STAT3. Thus, we connect this membrane channel protein with one of the most classical intracellular signaling pathways, and then provide a new direction to explain its biological functions. Enrichment analyses also suggested potential links between AQP9 and inflammatory or immune-related pathways, although these hypotheses require further experimental validation. In addition, the enrichment analysis shows that the PI3K-AKT pathway and EMT pathway are activated in the AQP9-high group; both signaling pathways collaborate with the JAK/STAT pathway [47,48]. Collectively, the JAK/STAT pathway might be the central pathway through which AQP9 promotes the malignant characteristics of ccRCC. By introducing the JAK/STAT pathway inhibitor Stattic in rescue experiments, we further confirmed the crucial role of this pathway in AQP9-mediated EMT and metastasis, thereby providing a potential therapeutic target for targeted interventions.

Given that AQP9 exerts pro-tumor effects and acts as a potential prognostic indicator in several other malignancies, the search for specific AQP9-targeted agents is likely to represent an effective therapeutic strategy.

Virtual screening is a computational technique that uses models and algorithms to prioritize candidate compounds from large chemical libraries that are most likely to be active against a specific biological target [49]. In recent years, drug repurposing strategies have attracted considerable attention because of their advantages of shorter development cycles, lower cost, and already established safety profiles [28]. Based on these new concepts and strategies in drug development, we selected an FDA-approved compound library in the present study and applied a hierarchical virtual screening approach to identify compounds with high binding affinity to AQP9. Virtual screening, molecular dynamics simulations, and a CETSA consistently identified Tedizolid as a stable and direct binder of AQP9. These results provided structural and thermodynamic support for the subsequent functional experiments. Recent studies have demonstrated that certain antibiotics exhibit direct anti-tumor activity across various cancer types, supporting the potential repurposing of Tedizolid in ccRCC [50,51,52,53].

In our further functional experiments, we observed Tedizolid could inhibit ccRCC cells’ viability in a concentration-dependent way; at the same time, it decreased the level of the protein AQP9. Its IC50 value was 13.48 μM and 18.15 μM, respectively, for those two ccRCC cell lines mentioned above, indicating its favorable efficacy in vitro. We also found that Tedizolid treatment reversed the malignant changes and the JAK/STAT pathway activation caused by AQP9 overexpression. These results suggest that Tedizolid has anti-tumor effects by targeting the AQP9-JAK/STAT axis.

We have proved the above conclusions in another way closer to clinical use: a subcutaneous tumor model was made in nude mice. And the results also matched our previous cell experiment results: overexpressed AQP9 could make the tumor grow faster, while treatment with Tedizolid could inhibit this process. These findings support that Tedizolid could reverse the oncogenic effect of AQP9 in ccRCC. In addition, immunohistochemical analyses of the tumor tissues provided direct in vivo evidence for AQP9-regulated molecular pathways. In comparison to the control group, the tumors with AQP9 overexpression showed significantly elevated protein levels of p-STAT3, N-cadherin, Vimentin, MMP2 and MMP9. However, after treatment with Tedizolid, their expression levels returned to near normal ranges. These in vivo data validated, at the whole-organism level, the central mechanism that “AQP9 drives ccRCC progression by promoting EMT through activation of the JAK/STAT3 pathway” and ultimately demonstrated that Tedizolid exerts anti-tumor effects by targeting this axis. Furthermore, from the perspective of clinical application, Tedizolid is an approved oxazolidinone antibiotic with well-characterized pharmacokinetic and safety profiles in humans based on long-term clinical use [54], which affords substantial advantages for its rapid repurposing as a therapeutic agent for ccRCC.

Although this study provides several valuable insights, it also has certain limitations. First, the preliminary evidence regarding the prognostic value of AQP9 mainly derives from public databases; although we validated its protein expression using tissue samples from our center, larger cohorts from our institution and multivariate survival analyses are still needed to provide more direct clinical evidence. Second, although we clarified the downstream effects of AQP9-mediated activation of the JAK/STAT pathway, the specific upstream molecular events through which this channel protein triggers the signaling cascade (for example, whether it acts by modulating the transport of particular substrates) remain unclear, representing an important scientific question for future investigation. Third, the qRT-PCR data in this study were normalized using only β-actin as the housekeeping gene. Although preliminary experiments confirmed that β-actin expression remained stable under our experimental conditions, the inclusion of an additional reference gene (e.g., GAPDH or 18S rRNA) would provide further validation, and this represents a limitation of the present study. Fourth, regarding the mechanistic relationship between AQP9 and the JAK/STAT pathway, our conclusion that AQP9-driven EMT and metastasis are dependent on this signaling axis is currently supported by pharmacological inhibition experiments using Stattic. While Stattic is a well-characterized selective STAT3 inhibitor and pharmacological rescue is a widely accepted approach for pathway validation, genetic rescue experiments (e.g., restoration of constitutively active STAT3 in AQP9-knockdown cells) would provide a more stringent test of causality. This represents an important direction for future investigation. Fifth, the molecular mechanism by which Tedizolid binding leads to reduced AQP9 protein abundance remains to be elucidated. In addition, whether this reduction is a direct consequence of Tedizolid binding or an indirect effect secondary to pathway inhibition has not been determined in the present study.

In this study, we identified AQP9-mediated activation of the JAK/STAT pathway as an important mechanism driving ccRCC progression and successfully repurposed the antibiotic Tedizolid as an AQP9-targeted inhibitor, thereby providing a new therapeutic strategy for ccRCC, particularly for refractory disease. Notably, despite the clear therapeutic effects and target modulation observed in animal models, the clinical efficacy of Tedizolid in ccRCC patients, its optimal dosing regimen, and potential off-target effects still need to be clarified by future studies.

## 4. Materials and Methods

### 4.1. Bioinformatics Analysis

Using The Cancer Genome Atlas (TCGA) database (https://portal.gdc.cancer.gov/), we analyzed the differential expression of AQP9 between ccRCC and normal tissues, evaluated its diagnostic potential in ccRCC, examined its association with various clinicopathological features, and assessed its relationship with patient prognosis. The capability of AQP9 to serve as an independent prognostic factor was further investigated via Cox multivariate regression analysis. Additionally, a KEGG enrichment analysis and a Gene Set Enrichment Analysis (GSEA) of AQP9-related genes were performed using the DAVID (https://davidbioinformatics.nih.gov) and MSigDB (https://www.gsea-msigdb.org/gsea/msigdb (accessed on 1 May 2026)) databases.

### 4.2. Clinical Specimens

Paired ccRCC and adjacent normal tissue samples (*n* = 10) were collected in 2025 from patients diagnosed with pathologically confirmed ccRCC at the First Affiliated Hospital of Dalian Medical University. This study was approved by the hospital’s Ethics Committee (Approval No. PJ-KS-KY-2025-1109). The inclusion criterion was patients with pathologically confirmed ccRCC who underwent standard sterile surgical procedures. Exclusion criteria included: (1) patients with an uncertain diagnosis, such as renal masses with indeterminate pathology; and (2) patients with other concurrent malignancies. All tissue specimens were preserved at −80 °C until further analysis.

### 4.3. Western Blot

Total cellular proteins were extracted using RIPA lysis buffer (Beyotime, Shanghai, China). The samples were resolved by SDS-PAGE, transferred onto PVDF membranes, and then incubated overnight at 4 °C with primary antibodies against AQP9 (sc-74409 G-3, 1:100, Santa Cruz Biotechnology, Dallas, TX, USA)), N-cadherin (A0433, 1:1000, ABclonal, Wuhan, China), E-cadherin (A0433, 1:1000, ABclonal, China), Vimentin (10366-1-AP, 1:20,000, Proteintech, Rosemont, IL, USA), Snail (A5243, 1:1000, ABclonal, China), MMP2 (A19080, 1:1000, ABclonal, China), MMP9 (F0008, 1:1000, Selleck Chemicals, Houston, TX, USA), phosphorylated JAK2 (AP0531, 1:1000, ABclonal, China), JAK2 (K5N8, 1:1000, Selleck, WA, USA), phosphorylated STAT3 (B17F7, 1:2000, Selleck, WA, USA), STAT3 (WL01836, 1:1000, Wanleibio, Shenyang, China), and β-actin (AC038, 1:20,000, ABclonal, China). Next, the membranes were incubated with HRP-conjugated secondary antibodies at room temperature for 1 h. Protein signals were detected using an ECL chemiluminescence detection kit (Beyotime, China). To minimize inter-membrane variability (e.g., background or exposure differences), data from each independent biological replicate (each membrane) were normalized by setting the mean “target/control” ratio of the control group on that membrane to 1. All individual values (both control and experimental groups) on the same membrane were then divided by this reference value to obtain normalized relative expression levels (fold change). The normalized data from three independent replicates were combined for statistical analysis.

### 4.4. Cell Culture and Transfection

Human renal cell carcinoma cell lines (786-O, A498, 769P, and Caki-1) and the human renal proximal tubular epithelial cell line (HK-2) were obtained from the American Type Culture Collection (ATCC, Manassas, VA, USA) [55,56,57] and were authenticated by short tandem repeat profiling. The cells were cultured under 5% CO_2_ in humidified air at 37 °C. In order to regulate gene expression, we purchased lentivirus particles from (Genechem, Shanghai, China) China, produced stable transfected cell lines according to the manufacturer’s guidance, and used blank vector transductants as controls. All the cell lines used in this research were mycoplasma-free. For the JAK/STAT pathway inhibition experiments, the cells were treated with Stattic (Selleck, WA, USA) at a single concentration of 10 μM. The rationale is as follows: (1) preliminary Western blot analysis confirmed that 10 μM Stattic effectively suppressed p-STAT3 expression in both 786-O and Caki-1 cells after 48 h; (2) the purpose of the rescue experiment was to verify pathway involvement rather than to generate a full dose–response curve. Therefore, a single, functionally validated concentration is justified.

### 4.5. Quantitative Real-Time Polymerase Chain Reaction (qRT-PCR)

Total RNA was extracted from the cells using TRIzol reagent (Thermo Fisher Scientific, Waltham, MA, USA). RNA was reverse-transcribed into cDNA using the HiScript Q RT SuperMix for qPCR (Vazyme, Nanjing, China). qPCR was performed by mixing cDNA template with primers and ChamQ Universal SYBR qPCR Master Mix (Vazyme, China) according to the manufacturer’s instructions, using a Bio-Rad real-time PCR detection system. β-actin was selected as the housekeeping gene for qRT-PCR normalization. Preliminary experiments showed that the Ct values of β-actin remained stable across the different treatment groups in ccRCC cell lines (786-O and Caki-1) and tissue samples (coefficient of variation < 5%), indicating that its expression was not affected by our experimental conditions. Additionally, previous studies have supported the use of β-actin as a reliable housekeeping gene in ccRCC research [58]. The primer sequences were as follows (synthesized by Genecreate, Wuhan, China):

AQP9—Forward: 5′-CCCAGCTGTGTCTTTAGCAATG-3′;

AQP9—Reverse: 5′-TTTCCACCAGCAAAGGACATAA-3′;

β-actin—Forward: 5′-CTCCATCCTGGCCTCGCTGT-3′;

β-actin—Reverse: 5′-GCTGTCACCTTCACCGTTCC-3′.

### 4.6. CCK-8 Assay

The cells were seeded in 96-well plates at a density of 2 × 10^3^ cells per well. After cell attachment, 90 μL of serum-free medium and 10 μL of CCK-8 (Cell Counting Kit-8) reagent (APExBIO Technology LLC, Houston, TX, USA) were added to each well at 0, 24, 48, and 72 h. The plates were then incubated for 1.5 h at 37 °C in a humidified atmosphere containing 5% CO_2_. Absorbance was measured at 450 nm using a microplate reader. Wells containing only medium and CCK-8 reagent without cells served as blanks for background correction.

### 4.7. Colony Formation Assay

The cells were planted at low density in 6-well plates (1 × 10^3^ cells/well), then cultured until colonies appeared, which contained over 50 cells. The generated colonies were fixed with 4% paraformaldehyde at room temperature for 20 min first, then stained using 0.5% crystal violet solution (1 mL/well) again for 15 min. Photographs were finally taken as records of such colonies.

### 4.8. Wound Healing Assay

A total of 6 horizontal reference lines (3 lines per well) were drawn at the bottom of each 6-well plate before cell seeding. Then, the cells (5 × 10^5^/well) were grown up to around 90% confluence at 37 °C. Afterwards, a scratch was made parallel to those reference lines with the help of the tip of a pipette (200 µL). After washing three times with PBS buffer solution, new medium was poured into it. At last, images were taken at the zero hour mark, six-hour mark, and at the twelve-hour mark of the wound location via an optical microscope.

### 4.9. Migration and Invasion Assays

Cell migration was carried out by means of a Transwell chamber (8 μm pore size), which was not covered with Matrigel beforehand. As to the invasion test, we needed to coat the chamber first with Matrigel (Biosharp, Beijing, China). Specifically, Matrigel matrix was thawed overnight at 4 °C and was then diluted at a ratio of 1:8 with serum-free medium. We took 30 μL of the mix and put it on top of the chamber, and incubated at 37 °C for 1 h until gelling was completed; afterwards, we added cells (cell density was 1 × 10^5^, and volume was 200 μL serum-free medium) to the upper chamber. Meanwhile, in the lower chamber, there was already 800 μL of medium containing 10% FBS as an attractant substance. After culturing for another 24 h, these cells with migratory/invasive capability would appear on the underside. Next, they were firstly treated with 4% paraformaldehyde fixation solution for thirty minutes and then stained with 1% crystal violet for twenty minutes. Finally, pictures could be taken via optical microscopy.

### 4.10. Virtual Screening

A library of 2158 FDA-approved small molecules from the DrugBank database was prepared using the LigPrep module within the **Schrödinger Maestro 13.5 (2023.1)** molecular modeling suite. Ligand structures were optimized, and conformational states were generated at pH 7.4 using the OPLS4 force field. The three-dimensional structure of AQP9 was obtained from PDB and processed using Maestro. Binding pockets were identified and receptor grids were generated accordingly. Following Lipinski’s Rule of Five pre-filtering, a sequential virtual screening (HTVS → SP → XP docking) was performed. MM-GBSA calculations identified six candidate compounds with binding free energies (ΔG) below −30 kcal/mol, which were selected for further study based on their superior binding profiles.

### 4.11. Molecular Dynamics Simulation

A 100 ns molecular dynamics (MD) simulation of the AQP9–Tedizolid complex was performed using GROMACS 2025. The protein was modeled with the AMBER99SB-ILDN force field, and the ligand topology was generated using the GAFF2 force field. The protein and ligand topologies were merged, ensuring no atom type conflicts. The complex was solvated in a cubic box with TIP3P water molecules, maintaining a minimum distance of 1.2 nm between the complex and the box boundaries. Na^+^ and Cl^−^ ions were added to neutralize the system charge and achieve a physiological ion concentration of 0.15 M. The system was first energy-minimized. Subsequently, it was equilibrated under NVT (constant Number of particles, Volume, and Temperature) ensemble conditions for 2 ns (1,000,000 steps) at 310 K, followed by equilibration under NPT (constant Number of particles, Pressure, and Temperature) ensemble conditions for 2 ns (1,000,000 steps) at 310 K and 1 bar. Finally, a production MD simulation was run for 100 ns (50,000,000 steps, 2 fs step size) under NPT conditions (310 K, 1 bar). Trajectory frames were saved every 1000 steps for subsequent analysis. The following parameters were calculated using GROMACS tools: root mean square deviation (RMSD, for overall structural stability), root mean square fluctuation (RMSF, for residue flexibility), radius of gyration (Rg, for molecular compactness), solvent accessible surface area (SASA, for solvent exposure), number of protein–ligand hydrogen bonds (for interaction stability), 2D and 3D free energy landscapes, and the MM/PBSA binding free energy (including the average binding free energy and per-residue energy decomposition).

### 4.12. Cellular Thermal Shift Assay

The cells were treated with Tedizolid or DMSO for 2 h in a 37 °C, 5% CO_2_ incubator. After treatment, the cells were harvested by scraping and divided into seven equal aliquots (100 μL each). These aliquots were heated at gradient temperatures (40, 45, 50, 55, 60, 65, and 70 °C) for 3 min, then immediately chilled on ice for 5 min. Cell lysis was achieved by three cycles of freezing in liquid nitrogen and thawing. Subsequently, the lysates were centrifuged at 12,000× *g* for 15 min at 4 °C to separate the soluble (non-denatured) proteins from the insoluble (denatured) aggregates. The soluble fraction was mixed with loading buffer, and the amount of AQP9 was quantified by Western blot to assess its thermal stability upon ligand binding.

### 4.13. Cell Viability Assay

We seeded the cells onto 96-well plates with cell densities set at 6 × 10^3^ per well. When the cells were attached firmly, we treated them with full medium containing Tedizolid (0, 1, 5, 10, 15, 20, 25, and 30 μM; 856866-72-3, MedChemExpress, South Brunswick Township, NJ, USA) respectively. After another 48 h, we then added 90 μL of serum-free medium and 10 μL of CCK-8 solution (ApexBio, USA) into every well. Then we incubated them at 37 °C with humidified air containing 5% CO_2_ for about one and a half hours. Finally, we read out the OD values at a wavelength of 450 nm via microplate reader. Medium plus CCK-8 but lacking cells acted as a blank to deduct background values later. We performed the non-linear regression analysis (log(inhibitor) vs. response with variable slope) through the GraphPad Prism 10.0 program so as to obtain IC50 values.

### 4.14. Xenograft Tumor Model

Our animal experiment was approved by the Animal Ethics Committee of Dalian Medical University (approval number: L250916387). All the procedures were strictly followed according to institutional guidelines for animal studies.

Male BALB/c nude mice, at four weeks old, were randomly divided into three groups (*n* = 7 each): (1) LV-CON; (2) LV-AQP9; and (3) LV-AQP9 + Tedizolid. **They were then acclimatized for one week under specific pathogen-free (SPF) conditions (22 ± 1 °C, 55 ± 5% humidity, 12 h light/dark cycle) with free access to autoclaved food and water.** Tumor cell inoculation: The logarithmic phase of the LV-CON/786-O cell line and LV-AQP9/786-O cell line were then trypsinized and resuspended to a density of 4 × 10^7^/million in PBS. The cells were evenly mixed with Matrigel at ratio of 1:1 before injecting them subcutaneously (200 μL/mouse) into the right axillary region of the mice. Six days later, we could palpate the formed tumors. Next, Tedizolid (at 30 mg/kg, 200 μL/mouse) was given to the mice through an oral gavage at an interval of two days in the form of CMC-Na (0.5%) containing Tween-80 (0.1%) saline. The control group received vehicle solution (CMC-Na, 0.5%; Tween-80, 0.1%, in saline) in an equal amount. We used a vernier caliper to measure tumor sizes; then volume could be obtained using the following formula: V = 1/2 × (a × b^2^), where “a” represents the largest diameter and “b” means the smallest one of this tumor. Meanwhile, the body weights of all animals were recorded every day during the entire experiment process. Thirty days after tumor cell inoculation, the mice were killed, and the excision of tumors was performed. After weighing them, part of the tissues would be frozen rapidly in liquid nitrogen so as to analyze molecular-level information subsequently. Others were fixed with cold 4% paraformaldehyde in preparation for checking under the microscope about histological structure.

### 4.15. Immunohistochemical Staining

The tissues fixed in formaldehyde and then put into paraffin were sliced at a thickness of 4 µm and dried in an oven at 60 °C for two hours. Then, the slices were used to perform HE staining according to routine procedure and to perform an immunohistochemistry analysis. As regards the latter, after deparaffinization and rehydration, antigen retrieval was carried out using EDTA buffer. The next step was blocking endogenous peroxidase activity with 3% H_2_O_2_; the last was to avoid non-specific binding through incubation with 10% goat serum. The primary antibodies used were as follows: anti-AQP9 (catalog # A8540, ABclonal, Wuhan, China; 1:100 dilution), anti-p-STAT3 (catalog # AP0705, ABclonal Technology; 1:100 dilution), anti-N-cadherin (catalog # WL01047, Wanleibio, Shenyang, China; 1:200 dilution), anti-Vimentin (catalog # 10366-1-AP, Proteintech, Rosemont, IL, USA; 1:3000 dilution), anti-MMP2 (catalog # A19080, ABclonal Technology; 1:100 dilution), and anti-MMP9 (catalog # A0289, ABclonal Technology; 1:100 dilution). All the antibodies were incubated overnight at 4 °C. They were rinsed with PBS after that, then incubated with secondary antibodies conjugated to HRP at room temperature for one hour. Lastly, they were stained with DAB chromagen and counterstained with Hematoxylin. The final score was calculated by combining the percentage of positive cells (0: <10%; 1: 10–25%; 2: 26–50%; 3: 51–75%; and 4: 76–100%) and staining intensity (0: negative; 1: weak; 2: moderate; and 3: strong).

### 4.16. Statistical Analysis

Statistical analyses were conducted on the data from at least three independent biological replicates and were expressed as mean ± SD. GraphPad Prism 10.0 (GraphPad Software, Boston, MA, USA) was used for data visualization, while SPSS 26.0 (SPSS Inc., Chicago, IL, USA) handled the advanced analyses. Normality and homogeneity of variance were assessed (e.g., Shapiro–Wilk test) to select appropriate tests. For two-group comparisons meeting parametric criteria, Student’s *t*-test was applied. Multiple group comparisons used one-way ANOVA with Tukey’s post hoc test if assumptions were met; otherwise, the Kruskal–Wallis test followed by Dunn’s post hoc test was used. Tumor volume measurements were analyzed by two-way repeated measures ANOVA. Categorical data were analyzed by a Chi-square test. A two-sided *p*-value of less than 0.05 was considered statistically significant for all tests. The significance levels in the figures were denoted as follows: * *p* < 0.05, ** *p* < 0.01, *** *p* < 0.001, **** *p* < 0.0001 and ns indicated no significance.

## 5. Conclusions

**Figure 10:**
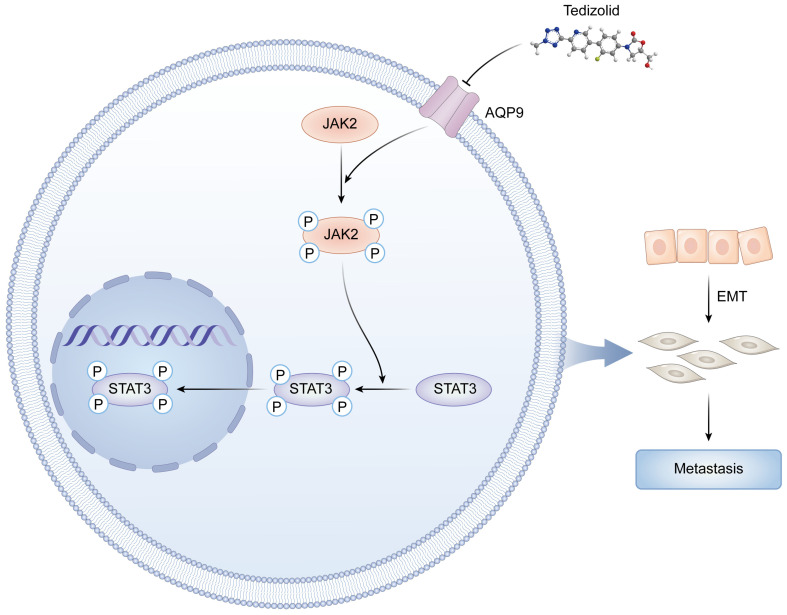
A mechanistic model of Tedizolid targeting the AQP9-JAK/STAT axis to suppress metastasis in ccRCC.

In conclusion, this study establishes the role of AQP9 as a key oncogenic driver in ccRCC. Mechanistically, our data indicate that AQP9-driven metastatic progression is dependent on JAK/STAT signaling, as the pharmacological inhibition of STAT3 partially attenuates AQP9-mediated EMT, migration, and invasion. Furthermore, through virtual screening, we identified Tedizolid as an effective AQP9 inhibitor, which suppresses the activity of the JAK/STAT pathway by downregulating AQP9 expression, thereby inhibiting the proliferation, migration, and invasion of ccRCC cells (Figure 10). Overall, these findings suggest that the Tedizolid/AQP9/JAK/STAT axis may represent a novel therapeutic strategy for the treatment of advanced ccRCC.

## Figures and Tables

**Table 1:** Multivariate Cox regression analysis of OS in TCGA cohort.

Covariate	HR (95%CI)	*p* Value
**AQP9**	**1.712 (1.025–2.859)**	**0.04**
Age	1.745 (1.042–2.922)	0.034
T Stage	1.429 (0.574–3.557)	0.443
N Stage	0.481 (0.201–1.151)	0.1
M Stage	2.199 (1.177–4.108)	0.014
AJCC Stage	0.745 (0.258–2.151)	0.586
Grade	1.566 (0.896–2.737)	0.115
Ragnum Hypoxia Score	1.046 (1.003–1.09)	0.036
Aneuploidy Score	0.978 (0.947–1.009)	0.166
Person Neoplasm Cancer Status	4.163 (2.389–7.252)	<0.001
Gender	1.253 (0.746–2.105)	0.394

**Bold font indicates the primary variable of interest (AQP9).**

**Table 2:** Multivariate Cox regression analysis of PFS in TCGA cohort.

Covariate	HR (95%CI)	*p* Value
**AQP9**	**2.543 (1.068–6.054)**	**0.035**
Age	0.539 (0.222–1.31)	0.173
T Stage	1.86 (0.599–5.776)	0.283
N Stage	0.182 (0.05–0.667)	0.01
M Stage	0.745 (0.301–1.84)	0.523
AJCC Stage	1.981 (0.456–8.609)	0.362
ISUP Grade	1.635 (0.693–3.859)	0.262
Ragnum Hypoxia Score	1.087 (1.025–1.153)	0.005
Aneuploidy Score	0.975 (0.93–1.023)	0.302
Person Neoplasm Cancer Status	16.326 (5.939–44.88)	<0.001
MSI MANTIS Score	1.8 × 10^−7^ (1.6 × 10^−7^–0.192)	0.028

**Bold font indicates the primary variable of interest (AQP9).**

**Table 3:** Multivariate Cox regression analysis of DSS in TCGA cohort.

Covariate	HR (95%CI)	*p* Value
**AQP9**	**1.999 (1.029–3.883)**	**0.041**
Age	1.006 (0.487–2.078)	0.986
T Stage	1.407 (0.566–3.498)	0.462
N Stage	0.408 (0.168–0.992)	0.048
M Stage	1.637 (0.839–3.192)	0.148
AJCC Stage	1.375 (0.399–4.74)	0.614
Grade	1.354 (0.618–2.967)	0.449
Ragnum Hypoxia Score	1.067 (1.021–1.115)	0.004
Aneuploidy Score	0.966 (0.93–1.003)	0.071
Person Neoplasm Cancer Status	25.883 (8.794–76.185)	<0.001
Gender	1.159 (0.583–2.304)	0.674

**Bold font indicates the primary variable of interest (AQP9).**

## Data Availability

The raw data supporting the conclusions of this article will be made available by the authors on request.

## Abbreviations

The following abbreviations are used in this manuscript:

ccRCC	Clear Cell Renal Cell Carcinoma
RCC	Renal Cell Carcinoma
AQP9	Aquaporin 9
EMT	Epithelial–Mesenchymal Transition
JAK/STAT	Janus Kinase-Signal Transducer and Activator of Transcription
TCGA	The Cancer Genome Atlas
KEGG	Kyoto Encyclopedia of Genes and Genomes
GSEA	Gene Set Enrichment Analysis
qRT-PCR	Quantitative Real-Time Polymerase Chain Reaction
CCK-8	Cell Counting Kit-8
CETSA	Cellular Thermal Shift Assay
MD	Molecular Dynamics
RMSD	Root Mean Square Deviation
RMSF	Root Mean Square Fluctuation
Rg	Radius of Gyration
SASA	Solvent Accessible Surface Area
MM/GBSA	Molecular Mechanics/Generalized Born Surface Area
IC50	Half Maximal Inhibitory Concentration
OS	Overall Survival
PFS	Progression-Free Survival
DSS	Disease-Specific Survival
HIF	Hypoxia-Inducible Factor
VHL	Von Hippel-Lindau
MMP	Matrix Metalloproteinase
ECM	Extracellular Matrix
shRNA	Short Hairpin RNA
OD	Optical Density
PC	Principal Component
Tm	Melting Temperature
FDA	Food and Drug Administration
